# Exogenous Melatonin Alleviates Alkaline Stress by Removing Reactive Oxygen Species and Promoting Antioxidant Defence in Rice Seedlings

**DOI:** 10.3389/fpls.2022.849553

**Published:** 2022-03-09

**Authors:** Xuping Lu, Weifang Min, Yafei Shi, Lei Tian, Peifu Li, Tianli Ma, Yinxia Zhang, Chengke Luo

**Affiliations:** Agricultural College, Ningxia University, Yinchuan, China

**Keywords:** rice, alkaline stress, melatonin, reactive oxygen species, antioxidant system

## Abstract

Saline-alkali stress seriously restricts rice growth, development, and production in northern China. The damage of alkaline stress on rice is much greater than that of salt due to ion toxicity, osmotic stress, and especially high pH. As a signal molecule, melatonin (N-acetyl-5-methoxytryptamine, MT) mediates many physiological processes in rice and participates in protecting rice from abiotic stress. The potential mechanism of exogenous melatonin-mediated alkaline stress tolerance is still largely unknown. In this study, the effects of melatonin on the morphological change, physiological property, and corresponding genes expression in rice seedlings were analyzed under alkaline stress (20 mmol L^−1^, pH 9.55). The results showed that the expression levels of MT synthesis genes (*TDC2*, *T5H*, *SNAT*, *ASMT1*, and *ASMT2*) were induced by both exogenous MT and alkaline stress treatment. The cell membrane was protected by MT, and the MT furtherly play role in scavenging reactive oxygen species (ROS), reducing lipoxygenase (LOX) activity, and malondialdehyde (MDA) content. The scavenging of ROS by melatonin is attributed to the coupling of the improvement of redox homeostasis and the enhancement of antioxidant enzyme activity and antioxidant content by upregulating the transcriptional levels of antioxidase genes. In the meantime, MT pretreatment promoted the accumulation of free proline, sucrose, and fructose by regulating the *OsP5CS*, *OsSUS7*, and *OsSPS1* gene expression level and increased chlorophyll content upregulating the expression of chlorophyll synthesis-related genes. Ultimately, the alleviating effect of exogenous melatonin on alkaline stress was reflected in increasing the leaf relative water content (RWC) and root-shoot ratio and reducing the leaf tip wilt index (TWI) through a series of physiological and biochemical changes. Melatonin pretreatment changed the expression level of MT synthesis genes which might contribute to MT synthesis in rice, consequently, activated the ROS scavenging system and alleviating the damage of alkaline stress on rice seedlings. Our study comprehensively understands the alleviating effect of exogenous melatonin on rice under alkaline stress.

## Introduction

Soil salinization is one of the important limiting factors affecting the sustainable development of agriculture (Shabala, 2013; Shahbaz and Ashraf, 2013). Approximately 7.6% of the world’s land (1 billion km^2^) is polluted by salinity and alkalinity. However, there are no effective methods to inhibit its expansion (Correa-Ferreira et al., 2019). The main harmful salts in saline-alkali soil include NaCl, Na_2_SO_4_, NaHCO_3_, and Na_2_CO_3_ (Yang et al., 2007). Usually, the stress induced by neutral salts NaCl and Na_2_SO_4_ and alkaline salt Na_2_CO_3_ and NaHCO_3_ is called salt stress and alkaline stress. In contrast, mixed saline-alkaline stress is caused by both neutral and alkaline salt (Lu et al., 2021). Although research on these topics has been conducted, most of them employed neutral salts to simulate saline-alkaline stress, and few studies have been carried out on alkaline salt stress. Osmotic stress and ion toxicity are the main damages of salt stress on plants (Flowers and Colmer, 2008). Alkaline stress causes osmotic stress and ion toxicity in the same way as salt stress does. More importantly, alkaline stress is caused mainly due to the solidification of nutrient elements in soil at a high pH value, which seriously interferes with the absorption of mineral nutrition by plant roots, leading to nutrient imbalance in plants, disorder of metabolism, and destruction of ion balance (Guo et al., 2017; Zhang et al., 2017a).

Rice (*Oryza sativa* L.) is the major food crop in many countries or regions, and its growth and development are hampered by abiotic stress (Zhang et al., 2013). As a salt-alkali sensitive crop, rice mortality gradually increases, with salt concentration, especially seedlings (Chunthaburee et al., 2016).

The plants’ growth, physiological, and biochemical metabolism are seriously disturbed by alkaline stress. The accumulation of inorganic ions in the soil raises the osmotic pressure causing osmotic stress and physiological drought in plants. Hence to maintain intracellular water potential stability, usually, plant cells synthesize and accumulate a variety of small molecular organic compounds such as proline, soluble protein, betaine, sugar, polyols, and polyamines (Sun et al., 2019). The high pH stress accompanied by alkaline stress leads to the solidification of organic elements such as carbon, nitrogen, phosphorus, and sulfur, thereby reducing the nutrient absorption and circulation in plants, damaging the roots of plants, and resulting in the loss of normal physiological functions of root cells (Robin et al., 2016; Neina, 2019). Under normal conditions, reactive oxygen species (ROS) produced by plants are important signaling molecules involved in plant growth and development, stress response, and other physiological activities (Baxter et al., 2014). However, under stress, a considerable amount of ROS is induced in plants, including hydrogen peroxide (H_2_O_2_), hydroxyl radical (OH^−^), superoxide radical (O_2_^·–^), and monolinear oxygen (^1^O_2_), causing oxidative damage of different cell components such as nucleic acid, protein, membrane, and carbohydrate (Ye et al., 2021). Simultaneously stress increases of lipoxygenase (LOX) activity, which accelerated the oxidation of polyunsaturated fatty acids (PUFAs) on the membrane catalyzed by LOX, increasing malondialdehyde (MDA) content (Kang et al., 2021). Plants have evolved enzymatic and non-enzymatic antioxidant systems to protect themselves from oxidative damage and maintained low-level ROS signals to maintain the steady state of ROS in organisms. These enzymatic antioxidant systems mainly include superoxide dismutase (SOD), peroxidase (POD), catalase (CAT), ascorbate peroxidase (APX), glutathione peroxidase (GPX), and glutathione reductase (GR; Wang et al., 2017a). SOD is the first line of defense in the plant antioxidant system, converting accumulated O_2_^·–^ into oxygen and H_2_O_2_, and then converting H_2_O_2_ into water and oxygen *via* CAT, APX, and POD (Luo et al., 2021). However, reduced ascorbic acid (ASA), reduced glutathione (GSH), carotenoids (Car), mannitol, and anthocyanins are non-enzymatic antioxidant systems which regulate intracellular ROS homeostasis (He et al., 2019).

Melatonin (N-acetyl-5-methoxytryptamine, MT) is a naturally occurring molecule and was first discovered in plants in 1995 (Dubbels et al., 1995). MT, as a plant growth regulator, not only regulates plant development but also alleviates the damage of abiotic and biotic stresses such as salt (Yan et al., 2021a), drought (Fleta-Soriano et al., 2017; Wang et al., 2017b), cold (Bajwa et al., 2014), light and high temperature (Tiryaki and Keles, 2012), heavy metals (Li et al., 2021a), dark- and heat-induced leaf senescence (Zhang et al., 2016, 2017b), nutrient deficiency (Kobylinska et al., 2018), and diseases (Byeon et al., 2016) on plants. MT is not only an efficient ROS scavenger but also interacts with ROS directly. Studies have shown that one molecule of MT can scavenge up to eight or more ROS through free radical scavenging reactions, allowing cell membrane to be stabilize and cellular oxidative stress to be reduce (Garcia et al., 2014; Yan et al., 2021a). On the other hand, by modulating the activity of antioxidant enzymes, MT can boost its antioxidant efficacy. Under abiotic stress, MT controls ROS accumulation by regulating antioxidant enzyme gene expression, activating antioxidant enzyme (including CAT, APX, POD, and SOD) activities (Nawaz et al., 2018; Al-Huqail et al., 2020).

So far, research on the effect of exogenous MT on rice stress tolerance has been focus on abiotic stressors such as low temperature (Li et al., 2021b), salt (Yan et al., 2020), and drought (Silalert and Pattanagul, 2021). However, there is relatively little data on exogenous MT’s ability to reduce alkaline stress. As per the previous studies, alkaline stress has a different effect on plants than salt stress. So, does exogenous MT have a comparable effect on rice physiological and biochemical processes under alkali stress as it does under other abiotic stresses? Furthermore, the underlying mechanisms of exogenous MT regulating rice response to alkaline stress are still unclear. Therefore, we investigated the effects of MT on ROS metabolism, osmotic adjustment substance accumulation, photosynthetic pigments, and antioxidant system, as well as gene expression changes related to MT synthesis and physiological metabolism of abiotic stress resistance in rice seedlings under alkaline stress. The purpose of this study is to help us better understand the molecular mechanism of exogenous MT in alleviating alkaline stress.

## Materials and Methods

### Plant Materials and Growth Conditions

Rice variety Zhonghua 11 (*Oryza sativa* L. cv. “Zhonghua No. 11”) was selected as the experimental material. The seeds were disinfected with 15% sodium hypochlorite for 5 min before rinsing with distilled water five times. The washed rice seeds were then placed in a 96-well hydroponic box containing rice nutrient solution. The plantlets were nurtured in the greenhouse after seed germination. The following were growth conditions in the greenhouse: natural sunlight, temperature 28°C/25°C (day/night), and photoperiod 14/10 h (day/night). Seedlings were incubated in rice nutrient solution throughout the culturing process, and the solution was periodically replaced every 3 days as per the method of Yan et al. (2020). Seedlings were utilized as experimental materials after reaching the three-leaf stage (about 21 days).

### Chemical and Alkaline Stress Treatments

Seedlings of the same size were selected for alkaline stress trials. Four different treatments were established for alkali stress experiments. Among these treatments, WC representatives double distilled water (ddH_2_O) pretreatment; WM representatives Melatonin (200 μmol L^−1^ MT pretreatment); AC representatives ddH_2_O pretreatment + alkali stress (20 mmol L^−1^, pH9.55 [NaHCO_3_:Na_2_CO_3_ = 1:1molar ratio)]; AM representatives MT pretreatment + alkali stress.

For WC and WM treatments, the seedling was sprayed separately with ddH_2_O and 200 μmol L^−1^ MT (20 ml per plate) on the leaf surface and then incubated in rice nutrient solution without stress. For AC and AM treatments, the seedlings were sprayed with either ddH_2_O or 200 μmol L^−1^ MT, and then grown in the rice nutrient solution containing 20 mmol L^−1^ NaHCO_3_/Na_2_CO_3_. The foliar spraying was done every day at 6:00 p.m. for 3 days before the alkaline stress treatment. The dose of MT was screened *via* preliminary testing with MT concentrations (100, 200, and 300 μmol L^−1^) and 200 μmol L^−1^ MT was found to be the most effective dose on the phenotype of rice seedlings under alkaline stress. After 5 days of varied treatments, leaf samples were collected, frozen in liquid nitrogen, and stored at −80°C until use. In the current study, four treatments (WC, WM, AC, and AM) were completely randomized block designs, repeated four times.

### Measurement of Growth and Physiological Parameters

#### Measurement of Leaf Tip Wilt Index, Relative Water Content, and Root-Shoot Ratio

The leaf tip wilt index (TWI) of rice seedlings was measured after 5 days of alkaline stress treatment using the following formula: TWI = *L*_1_/*L*_2_, where *L*_1_ and *L*_2_ are the length of leaf wilting part and total length of leaf, respectively.

The isolated leaves of the two strains of seedling were selected, the fresh weight (FW) of the isolated leaves was measured immediately and then immersed in deionized water in test tubes for 24 h. The water on the leaf surface was dried after the swelled leaf was withdrawn from the test tubes, and the turgid weight (TW) was calculated. Later, these leaves were dried at 70°C for 72 h, and their dry weight (DW) was recorded. RWC was calculated according to the following formula (Smart, 1974):


$$\mathrm{RWC}\;\left(\%\right)=\left[\left(\mathrm{FW}-\mathrm{DW}\right)/\left(\mathrm{TW}-\mathrm{DW}\right)\right]\times 100$$

The 15 strains of seedling in each treatment were randomly selected, the isolated roots and shoots of seedling were collected and dried at 70°C for 72 h, respectively, and then, theirs dry weight (DW) were recorded, respectively. Root-shoot ratio was calculated as the ratio of root DW to shoot DW (Xu et al., 2015).

#### Measurement of O_2_^·–^ and H_2_O_2_ Levels

Approach of Schneider and Schlegel (1981) was used to calculate the rate of O_2_^·−^ generation (Xu et al., 2015). Rice leaves (0.2 g) were ground in 4 ml, 65 mmol L^−1^ phosphate buffer (PBS, pH 7.8), and centrifuged at 5,000 *g* for 15 min at 4°C. Then, to 0.5 ml supernatant, 1 ml hydroxylamine hydrochloride, and 0.5 ml 65 mmol L^−1^ phosphate buffer were added, thoroughly mixed, and set aside for 1 h. One milliliter p-aminobenzenesulfonamide (17 mmol L^−1^) and 1 ml α-theanine (7 mmol L^−1^) were added to above mixture and incubated at 25°C for 20 min. The supernatant was measured spectrophotometrically at 530 nm after adding an equal volume of ether and centrifuging at 3,000 *g* for 3 min.

The methodology for measurement of H_2_O_2_ content was reported by Liu et al. (2010). Rice leaves (0.2 g) were chilled for 10 min before being homogenized in 4 ml 0.1% (*w*/*v*) trichloroacetic acid (TCA) and centrifuged at 12,000 *g*. Then, 0.2 ml ammonia and 0.1 ml 95% (*v*/*v*) hydrochloric acid solution containing 20% (*v*/*v*) TiC1_4_ were thoroughly mixed to 1 ml supernatant and again centrifuged at 10,000 *g* for 10 min at 4°C. After removing the supernatant, the deposit was washed continually with precooled acetone (−20°C) and dissolved in 3 ml of 1 mmol L^−1^ H_2_SO_4_. The absorbance of the resulting solution was recorded on a spectrophotometer at 410 nm.

#### Measurement of LOX and MDA Levels

Lipoxygenase activity was determined according to the method of Doderer et al. (1992). The absorbance value was taken using linoleic acid as a substrate at 234 nm. MDA content was determined using the Niu et al. (2017) approach. Rice leaves (0.2 g) were homogenated with 5 ml of 5% TCA solution and then was centrifuged at 3,000 *g* for 20 min. Further, a mixture formed by adding 2 ml 0.67% TBA (thiobarbituric acid) to 2 ml supernatant was incubated at 100°C for 30 min and then cooled immediately on ice before centrifugation at 3,000 *g* for 10 min. Absorbance was taken at 532, 600, and 450 nm, respectively.

#### Chlorophyll Extraction and Identification

Rice leaves (0.1 g) were crushed in liquid nitrogen, then soaked in 5 ml extracting solution (acetone: ethanol = 1:1, *v*/*v*) at 25°C for 24 h by shaking 2–3 times thoroughly. The reaction mixture was centrifuged at 5,000 rpm for 10 min. The absorbance was measured by a spectrophotometer at 663 and 645 nm. The chlorophyll content was counted as per the following formula: Chl *a* = 12.21 × OD_663_–2.81 × OD_645_, Chl *b* = 20.13 × OD_645_–5.03 × OD_663_, Chl = Chl *a* + Chl *b* (Lichtenthaler and Wellburn, 1983).

#### Antioxidant Enzyme Extraction and Activity Assays

Rice leaves (0.4 g) were ground into powder in liquid nitrogen. A mixture formed by adding 4 ml precooled 50 mmol L^−1^ phosphate buffer containing 0.1 mmol L^−1^ Na_2_EDTA and 1% (*w*/*v*) PVP to powder was homogenized and centrifuged at 4°C and 12,000 *g* for 20 min. The resulting supernatant was used to determine the activities of SOD, POD, CAT, and APX (Doderer et al., 1992).

For SOD activity, 100 μl supernatant was added to 50 mmol L^−1^ PBS (pH 7.8) containing 0.1 mmol L^−1^ EDTA, 13 mmol L^−1^ methionine, and 75 μmol L^−1^ NBT, and 100 μl 2 μmol L^−1^ riboflavin solution was additionally added to the reaction mixture to detect SOD activity at 560 nm (Beyer and Fridovich, 1987).

Peroxidase activity was determined by the maehly method (Maehly and Chance, 1954). 100 μl enzyme extract was added into the reaction solution containing 100 mmol L^−1^ PBS (pH 6.0), 20 mmol L^−1^ guaiacol, and 40 mmol L^−1^ H_2_O_2_ to generate a resulting solution. The absorbance in unit time was measured at 460 nm.

For CAT activity, 3 ml 0.15 mol L^−1^ PBS (pH 7.8) and 5 μl 30% H_2_O_2_ were added to 100 μl enzyme extract to form the resulting solution. The decrease of absorbance in unit time was recorded at 240 nm (Havir and McHale, 1987).

For APX activity, the reaction mixture contained 50 mM PBS (pH 7.0), 0.5 mM AsA, 0.1 mM EDTA, 0.1 mM H_2_O_2_, and 30 μl of enzyme extract in a final volume of 3 ml. The reaction was started by adding H_2_O_2_. The activity was calculated from the recorded decrease in absorbance at 290 nm for 1 min (Niu et al., 2017).

#### Estimation of AsA and DHA

The contents of AsA and DHA were determined according to Murshed et al. (2013). Rice leaves (0.5 g) were homogenized with 4 ml of 6% trichloroacetic acid (TCA, *w*/*v*) on the ice and centrifuged at 4°C, 16,000 *g* for 10 min. The obtained supernatant was used to measure AsA and total ascorbic acid composed of AsA and DHA.

For AsA, 200 μl supernatant was mixed with 3.8 ml reaction solution including 0.8 ml 200 mmol L^−1^ PBS (pH 7.4), 1 ml 10% TCA, 0.8 ml 42% phosphoric acid (H_3_PO_4_), 0.8 ml 4% (*w*/*v*) 2,2′-bipyridine, and 0.4 ml 3% (*w*/*v*) ferric chloride. After that, the resulting reaction solution was incubated at 42°C for 60 min; the absorbance was measured at 525 nm.

The method for determination of total ascorbic acid was as follows: 200 μl 6 mmol L^−1^ dithiothreitol (DTT) was added to 200 μl supernatant, and the resulting mixture was homogenized and incubated immediately at 42°C for 15 min. 200 μl 0.4% (*w*/*v*) N-ethylmaleimide (NEM) was added to the reaction mixture formed and placed at 25°C for 2 min. Subsequently, 0.8 ml 200 mmol L^−1^ PBS (pH 7.4), 1 ml 10% TCA, 0.8 ml 42% phosphoric acid (H_3_PO_4_), 0.8 ml 4% (*w*/*v*) 2,2′-bipyridine, and 0.4 ml 3% (*w*/*v*) ferric chloride were added to above prepared mixture and were thoroughly mixed and immovably placed at 42°C for 60 min. The absorbance of the resulting solution was measured at 525 nm. The contents of total ascorbic acid and AsA were calculated *via* a standard curve. However, DHA content was measured by calculating the difference in the mean value of total ascorbic acid and AsA. The reduced to oxidized ascorbic acid (AsA/DHA) ratio was equal to the value obtained *via* dividing AsA concentration by DHA concentration.

#### Measurement of Proline, Sucrose, and Fructose Levels

The proline content was determined using the method described by Benitez et al. (2016). 0.5 g rice leaves were leached in 5 ml 3% (*w*/*v*) sulfosalicylic acid solution for 10 min and centrifuged at 4,000 *g* for 20 min after the above crude extract (100°C) was cooled. The obtained supernatant (2 ml) was mixed with 2 ml acetic acid and 2 ml 2.5% (*w*/*v*) acidic ninhydrin reagent and incubated at 100°C for 30 min. After cooling, 4 ml toluene was added to the reaction mixture, then stirred, and immovably placed for a moment before being centrifuged at 3,000 *g* for 5 min. The absorbance of the supernatant was taken at 520 nm. Proline content was calculated *via* a standard curve.

Rice leaves (0.2 g) were homogenized in 2 ml 80% ethanol, rockily incubated at 80°C for 10 min. The sample was then centrifuged at 4,000 *g* for 10 min after cooling the above reaction mixture. The supernatant was used to determine sucrose and fructose content (Rosa et al., 2009). Sucrose was determined according to the method of Cardini et al. (1955); Fructose was determined by the method of Roe and Papadopoulos (1954).

#### RNA Extraction, cDNA Synthesis, and RT-qPCR Analysis

The MiniBEST Plant RNA Extraction Kit (TaKaRa, Dalian, China) was used to extract total RNA from rice seedling leaves in three biological replicates using four treatments (WC, WM, AC, and AM). NanoDrop 2000/2000c (Gene Company Limited, Hong Kong, China) spectrophotometer was used to detect the purity and concentration of total RNA. Samples with A260/A280 ratio of 1.8–2.2 and A260/A230 ratio of about 2.0 were selected for subsequent analysis. PrimeScript™ RT reagent Kit with gDNA Eraser (TaKaRa, Dalian, China) was used to remove genomic DNA contamination and synthesis of first-strand cDNA. The detailed experimental procedure was carried out according to the manufacturer’s instruction.

Real-time quantitative RT-PCR (qRT-PCR) was performed in a qTOWER3G Real-Time PCR System (Analytikjena, Germany) using TB Green® *Premix Ex Taq*™ II reagent (TaKaRa). The 20 μl reaction system consisted of 10 μl TB Green *Premix Ex Taq* II, 1.6 μl PCR Primer (10 μmol L^−1^), 2.0 μl template (cDNA), and 6.4 μl ddH_2_O. Reaction conditions: 95°C, 3 min, 40 cycles including 95°C, 10 s, 60°C, and 30 s, the melting curve was generated by changing the amplification temperature from 60°C to 95°C. Rice *OsActin* was used as the internal reference gene. The experiment was done in triplicates. The relative expression levels of target genes were determined using the 2^−ΔΔCT^ method (Livak and Schmittgen, 2001). All of the above experiments were carried out using the corresponding manufacturer’s instructions.

Based on the Rice Genome Annotation Project (http://rice.uga.edu/), all of RT-qPCR primers were designed using Primer Premier 5 (version5.0, PREMIER Biosoft International, United States). The gene-specific primer information is shown in Table 1.

**Table 1:** Genes examined and RT-qPCR primer sequences.

Gene symbol	RGAP ID	Primer sequence (5'–3'; forward/reverse)	Product size
*ALM1*	LOC_Os06g05110	CTGGCTGGGTTTGGCTTGT/TCGCCTGTCATCCTTGTAATC	158
*OsPOX1*	LOC_Os01g15830	TGCCTGTTGATGCTCTGCT/CCGCCTGTGCTACGATGGA	157
*OsCATC*	LOC_Os03g03910	ACAACCACTACGACGGCTTCA/CCTTGGCAATCACCACCTT	153
*OsAPx2*	LOC_Os07g49400	TTGTGAGTGGCGAGAAGGA/GGCGTAATCCGCAAAGAA	128
*OsMPG1*	LOC_Os01g62840	CAAGGGATTACATTACAGGC/TCAGGACCAATCAGACAGC	148
*OsLOX5*	LOC_Os03g49380	CTGACCCAAATACAGAAAGCA/GGGAGAACACCCTCAACAATA	136
*OsP5CS*	LOC_Os05g38150	AATGACAGTTTAGCAGGAC/ACCACTATACAACCCATCC	87
*OsSUS7*	LOC_Os04g17650	TACAGGCACCAGATCCTAC/CTGCTGCTTGATTCTTTGA	200
*OsSPS1*	LOC_Os01g69030	GGCACAGCAAGACACTCCC/CGCCACGAACTAGACCATG	134
*OsFd1*	LOC_Os08g01380	CAGGCGGAGGAGGAAGGGAT/TAGGCGTGGCAGGTGAGGAC	161
*OsFdC2*	LOC_Os03g48040	TCCCACAGGACCAGTACATT/TTTATCCGAACCGCACAGC	103
*ChlP*	LOC_Os02g51080	ATCCCCGACGACAAGATG/GTCGCACTTGGGGAACAC	102
*CS*	LOC_Os05g28200	AGGCTCCAGCTTCAACCAG/GGCGAATCTTCCATATGTCG	71
*OsCAO1*	LOC_Os10g41780	TGCTCATCAAGCCTTCCTTCAGGTG/GCGTTGTTCTTTGCATAGTCAGCTC	115
*OsCAO2*	LOC_Os10g41760	CTGCTCGTCAAGCCTTCCTCCTCCT/CCCTCATGTTGTTCTTGGCATGTTG	130
*TDC2*	LOC_Os07g25590	CAGAGTACCGACACCACCT/AACCCATAGCAAGGAACAA	104
*T5H*	LOC_Os12g16720	AACCGACGACAATCTCAAGGC/GCTCCGTCATCACCCACTCC	92
*SNAT*	LOC_Os05g40260	TAATCCGAACTTTGCTCCA/GTACCAGAACATGCCCTTG	128
*ASMT1*	LOC_Os09g17560	GCCAAGGCTCCCAGTAACAA/ACCTTTCCTCCAGCATCCC	179
*ASMT2*	LOC_Os10g02880	GGCACAACGTGTCCAAGAA/GCGAAGCAGTGGTGGTAGA	90
*OsActin*	LOC_Os03g50885	GACCTTCAACACCCCTGCTA/ACAGTGTGGCTGACACCATC	114

### Statistical Analysis

Statistical analysis of all data were performed by SPSS 24.0 (IBM, Armonk, NY, United States) software with single factor variance (ANOVA). The least significant difference method (LSD) was used for multiple comparisons. Statistical significance was defined as *p* < 0.05. The error bars in all graphs represent the standard error of the mean.

## Results

### Changes in Phenotype, Leaf Tip Wilt Index, Relative Water Content, and Root-Shoot Ratio

**Figure 1:**
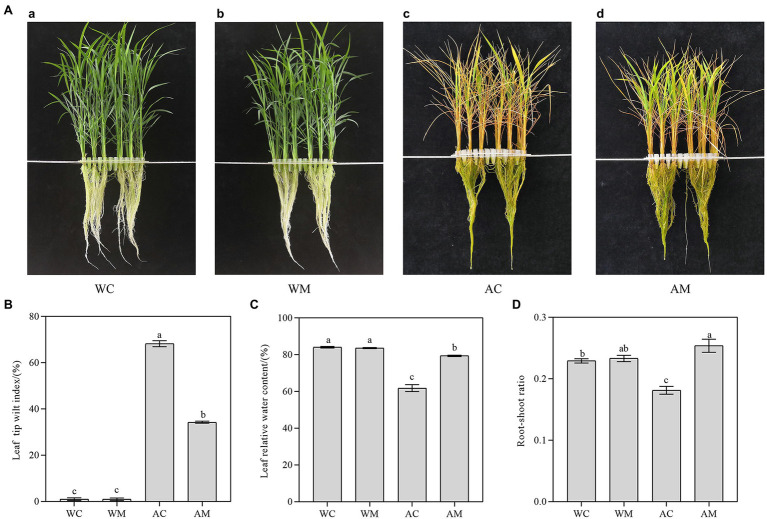
Melatonin alleviated alkaline stress to rice seedlings. **(A)** Growth performance of rice seedlings under different treatments; **(B)** leaf tip wilt index; **(C)** leaf water content; and **(D)** root-shoot ratio. Data represent means ± SEs of three replicate samples. Different letters denote significant differences (*n* = 3, and *p* < 0.05). WC, control; WM, control pretreated with MT; AC, alkaline stress; and AM, alkaline-stressed plants pretreated with MT.

Compared to the control (Figure 1Aa), exogenous MT did not alter the phenotype of rice seedlings in the WM treatment under normal growth conditions (Figure 1Ab). Under alkaline stress, the leaves of rice seedlings showed a large area of wilt and fewer green leaves (Figure 1Ac). However, MT pretreatment effectively alleviated the damage of alkaline stress on rice seedlings and maintained more green leaf area (Figure 1Ad).

Alkaline stress significantly influenced rice seedlings’ leaf tip wilt index (TWI). As shown in Figure 1B, the value of TWI was up to 68.18% in the AC treatment, whereas MT pretreatment remarkably decreased TWI (only 34.25%) in the AM treatment (*p* < 0.05). However, TWI decreased by 49.76% compared with that of AC treatment. In addition, MT pretreatment could also increase relative water content (RWC) and root-shoot ratio under alkaline stress. As shown in Figures 1C,D, RWC and the root-shoot ratio of AM treatment are significantly higher than that of AC treatment. Its RWC and root-shoot ratio are elevated by 28.32 and 40.02% relative to AC treatment.

### Changes in Chlorophyll Content and Chlorophyll Synthesis Gene Expression Levels

**Figure 2:**
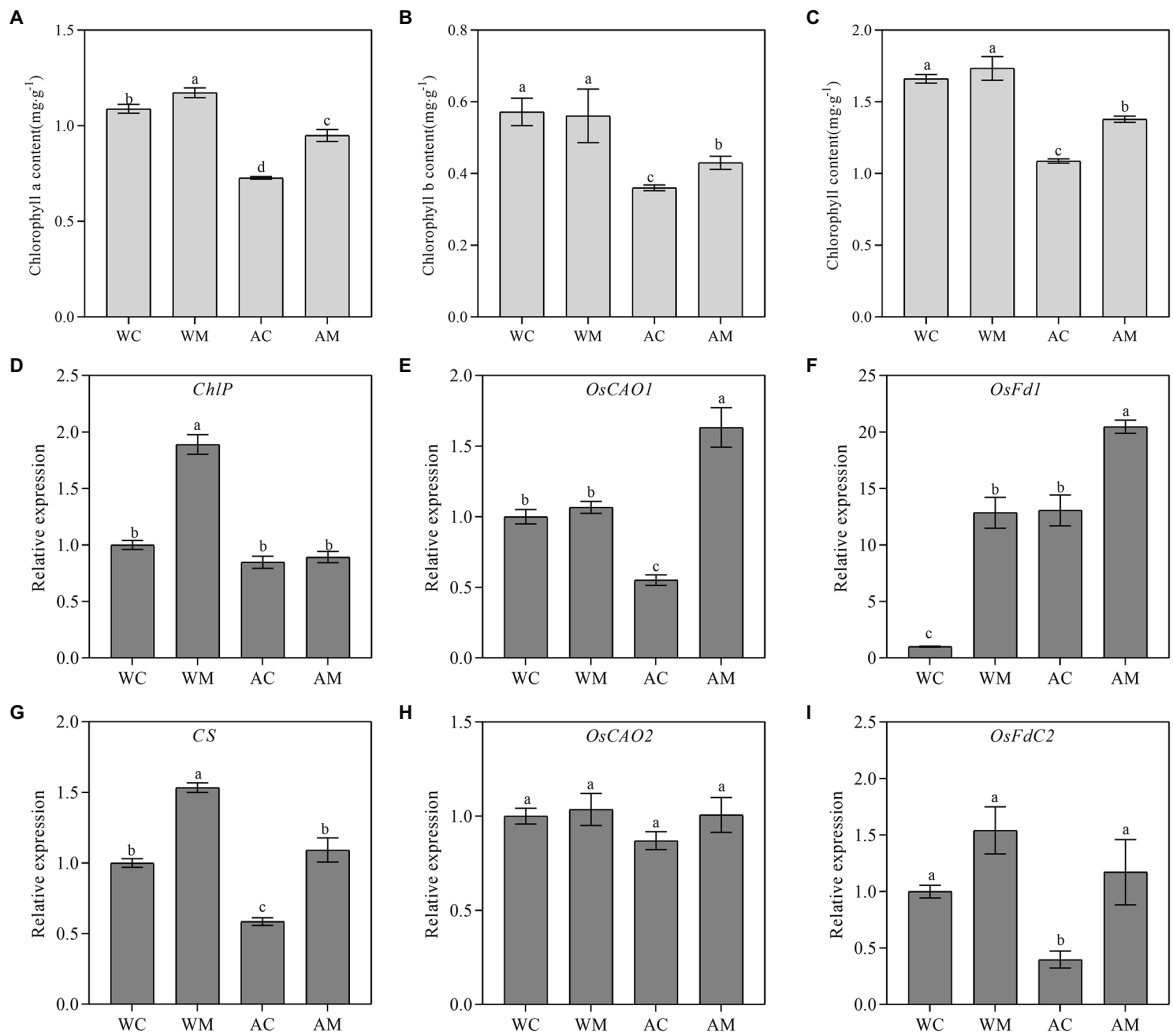
Effects of exogenous MT on chlorophyll content and chlorophyll synthesis-related genes. **(A)** Chlorophyll *a*; **(B)** Chlorophyll *b*; **(C)** Chlorophyll; and **(D–I)** Chlorophyll synthesis genes. Data represent means ± SEs of three replicate samples. Different letters denote significant differences (*n* = 3, and *p* < 0.05). WC, control; WM, control pretreated with MT; AC, alkaline stress; and AM, alkaline-stressed plants pretreated with MT.

Under normal growth conditions, only spraying MT increased the content of chlorophyll a (*p* < 0.05). The increased rate of chlorophyll a content was 7.71% compared with WC. In contrast, the chlorophyll a content of AC treatment decreased by 33.20% of WC treatment (*p* < 0.05) simultaneously. Interestingly, MT pretreatment significantly increases the chlorophyll a content by 30.58% in rice seedlings under alkaline stress compared with AC treatment (Figure 2A). For chlorophyll *b* and total chlorophyll content, there was no significant difference between WM and WC under normal growth conditions. However, both were significantly increased in AM treatment (*p* < 0.05) relative to AC treatment under alkaline stress, and their rate of increase reached 19.36 and 26.86%, respectively (Figures 2B,C).

*ChlP* and *CS* are mainly involved in synthesizing chlorophyll a. Exogenous MT significantly upregulated ChlP and CS expression levels under normal growth conditions (*p* < 0.05). The expression level of the *ChlP* gene displayed no obvious change in rice seedlings of AM than that of AC, whereas the relative expression level of *CS* in AM is markedly higher than that of AC under alkaline stress (Figures 2D,G).

*OsCAO1* and *OsCAO2* mainly participated in the synthesis of chlorophyll b. Exogenous MT has no significant effect on the transcriptional level of *OsCAO1* and *OsCAO2* under normal growth conditions (Figures 2E,H). However, under alkaline stress, MT pretreatment induced the expression of *OsCAO1* (Figure 2E), but it did not affect the expression of *OsCAO2* gene expression level (Figure 2H).

*OsFd1* and *OsFdC2* played an important role in photosynthetic electron transport and chlorophyll synthesis. MT pretreatment upregulated the relative expression levels of *OsFd1* and *OsFdC2* under normal growth conditions and alkaline stress. Under alkaline stress, exogenous MT significantly induced *OsFd1* gene expression level (Figures 2F,I).

### Changes in Reactive Oxygen Species Content

**Figure 3:**
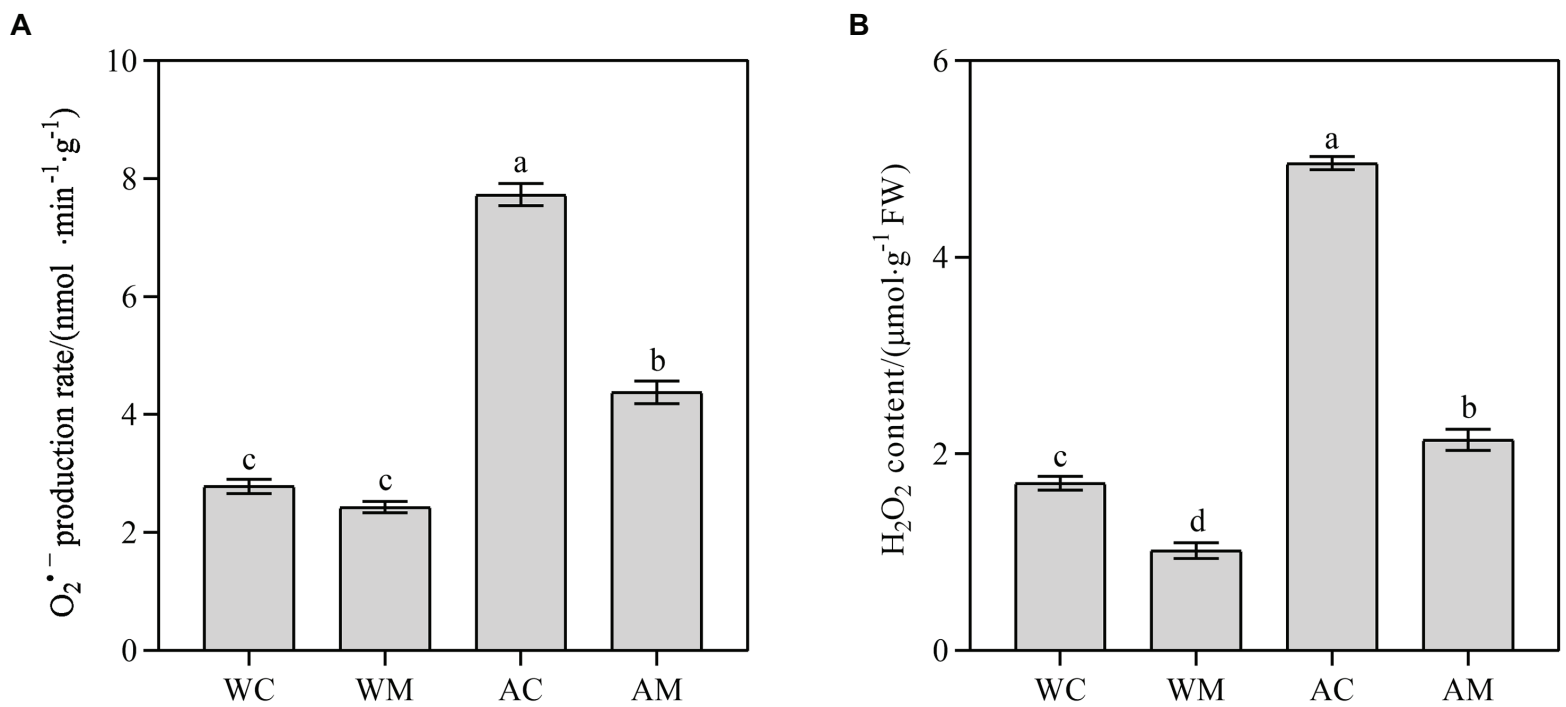
Effects of exogenous MT on O_2_^·–^ production rate and H_2_O_2_ content. **(A)** O_2_^·–^ production rate; **(B)** H_2_O_2_ content. Data represent means ± SEs of three replicate samples. Different letters denote significant differences (*n* = 3, and *p* < 0.05). WC, control; WM, control pretreated with MT; AC, alkaline stress; and AM, alkaline-stressed plants pretreated with MT.

Under normal growth conditions, only spraying MT decreased the content of H_2_O_2_ (*p* < 0.05; Figure 3B) but did not distinctly change the O_2_^·–^ production rate (Figure 3A). However, MT pretreatment reduces H_2_O_2_ content and O_2_^·–^ production rate under alkaline stress (Figures 3A,B). The reduction rate in AM treatment, respectively, accounted for 56.77 and 43.36% compared with AC. The results indicated that exogenous melatonin could effectively slow down the accumulation of ROS in rice under alkali stress.

### Changes of Lipoxygenase Activity, MDA Content, and *OsLOX5* Gene Expression Level

**Figure 4:**
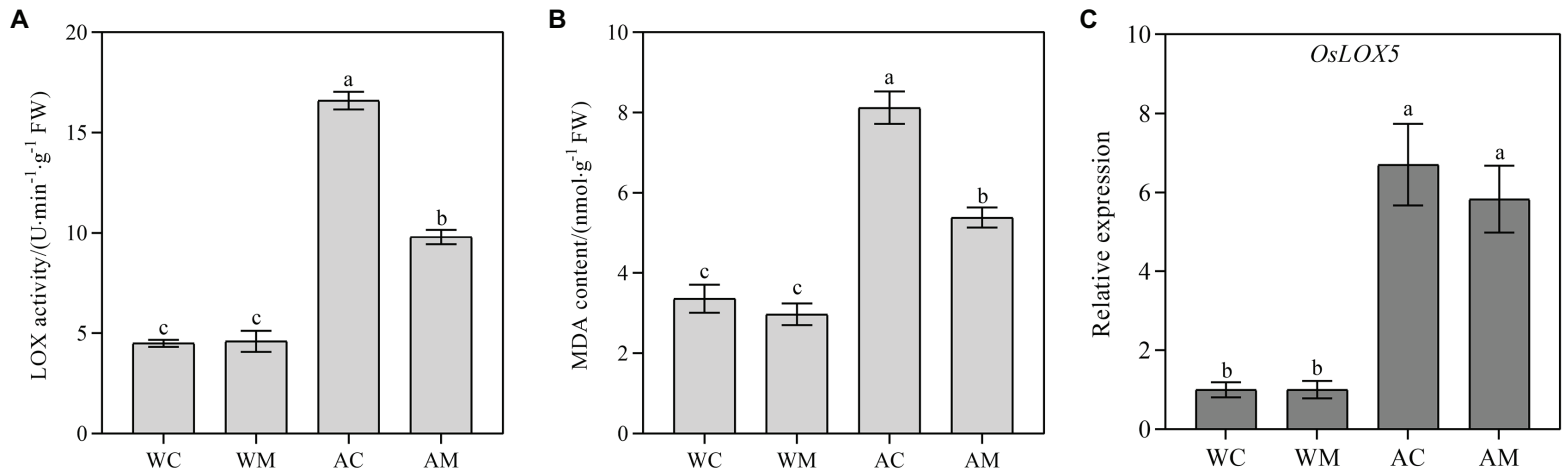
Effects of exogenous MT on lipoxygenase (LOX) activity, malondialdehyde (MDA) content, and *OsLOX5* expression. **(A)** LOX activity; **(B)** MDA content; and **(C)** Relative expression level of *OsLOX5* gene. Data represent means ± SEs of three replicate samples. Different letters denote significant differences (*n* = 3, and *p* < 0.05). WC, control; WM, control pretreated with MT; AC, alkaline stress; and AM, alkaline-stressed plants pretreated with MT.

Spraying MT had no obvious effect on LOX activity, the transcriptional level of *OsLOX5,* and MDA content under normal growth conditions (Figure 4). However, MT pretreatment significantly reduced LOX activity and MDA content under alkaline stress (Figures 4A,B). The reduction rate of AM treatment was 40.96 and 33.64% relative to AC treatment. Although exogenous melatonin downregulated *OsLOX5* gene expression under alkaline stress, its expression level did not change significantly between AM and AC (Figure 4C), suggesting that the *OsLOX5* gene may not be the key gene affecting lipoxygenase activity under alkali stress.

### Changes of Antioxidant Enzyme Activity and Antioxidase Gene Expression Levels

**Figure 5:**
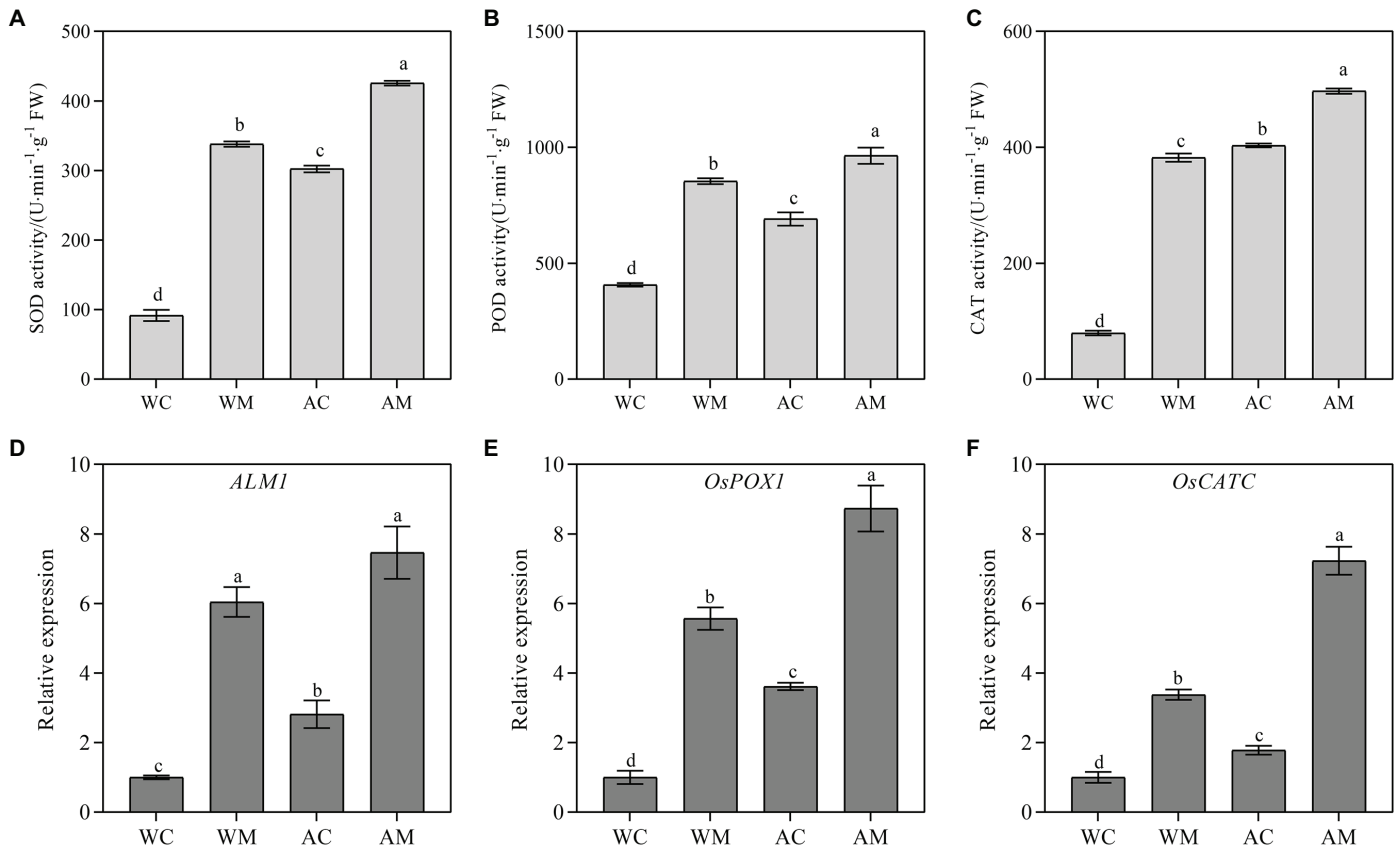
Effects of exogenous MT on antioxidase activity and antioxidant enzyme synthesis-related gene expression. **(A)** SOD activity; **(B)** POD activity; **(C)** CAT activity; and **(D–F)** *ALM1*, *OsPOX1*, and *OsCATC* relative expression level. Data represent means ± SEs of three replicate samples. Different letters denote significant differences (*n* = 3, and *p* < 0.05). WC, control; WM, control pretreated with MT; AC, alkaline stress; and AM, alkaline-stressed plants pretreated with MT.

Alkaline stress can increase the activities of antioxidant enzymes (SOD, POD, and CAT) in rice seedlings. Furthermore, MT pretreatment also significantly increases these three antioxidant enzymes activities under normal growth condition and alkaline stress (Figures 5A–C). *ALM1*, *OsPOX1*, and *OsCATC* are antioxidant genes selected, and we detected their expression levels. The results showed that exogenous MT could significantly upregulated three genes’ transcriptional levels under normal growth conditions and alkaline stress. The fold change of relative expression level under alkali stress was more obvious than normal (Figures 5D,E).

### Changes of APX Activity, ASA, DHA Content, and Their Encoding Gene Expression Level

**Figure 6:**
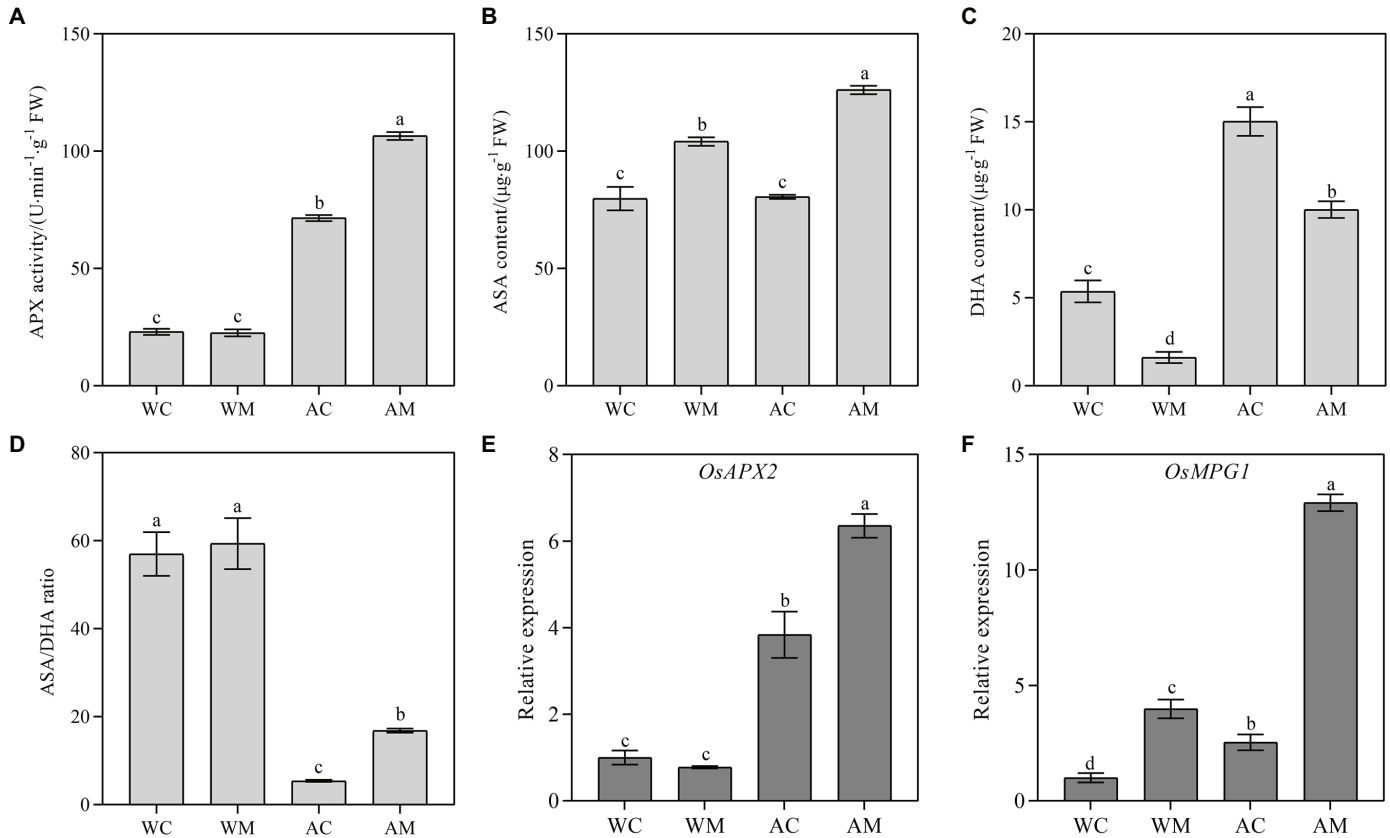
Effects of exogenous MT on APX activity, ASA, DHA content, and antioxidant-related genes. **(A)** APX activity; **(B)** ASA content; **(C)** DHA content; **(D)** ASA/DHA; and **(E,F)** *OsAPX2* and *OsMPG1* relative expression. Data represent means ± SEs of three replicate samples. Different letters denote significant differences (*n* = 3, and *p* < 0.05). WC, control; WM, control pretreated with MT; AC, alkaline stress; and AM, alkaline-stressed plants pretreated with MT.

Under normal growth conditions, spraying MT had no considerable influence on APX activity and the expression level of ascorbate peroxidases *OsAPX2* compared to the control (WC). In contrast, MT pretreatment significantly increased APX activity and upregulated *OsAPX2* gene expression level under alkaline stress (Figures 6A,E). Exogenous MT significantly increased ASA content and decreased DHA content inversely under both normal growth condition and alkaline stress (Figures 6B,C). For ASA/DHA ratio, there was no significant change between WM and WC treatment; however, under alkaline stress, exogenous MT affected its increase in AM treatment relative to AC treatment (Figure 6D). *OsMPG1* encodes GDP-D-mannan pyrophosphorylase (GMPase), which catalyzed GDP-D-mannan synthesis. GDP-D-mannan is a precursor to ascorbic acid (AsA) synthesis. As is shown in Figure 6F, exogenous MT significantly upregulated the transcriptional level of *OsMPG1* under both normal growth condition and alkaline stress. Exogenous MT strongly induced its expression level, especially under alkali stress.

### Changes in Free Proline, Sucrose, Fructose Content, and Their Related Gene Expression Levels

**Figure 7:**
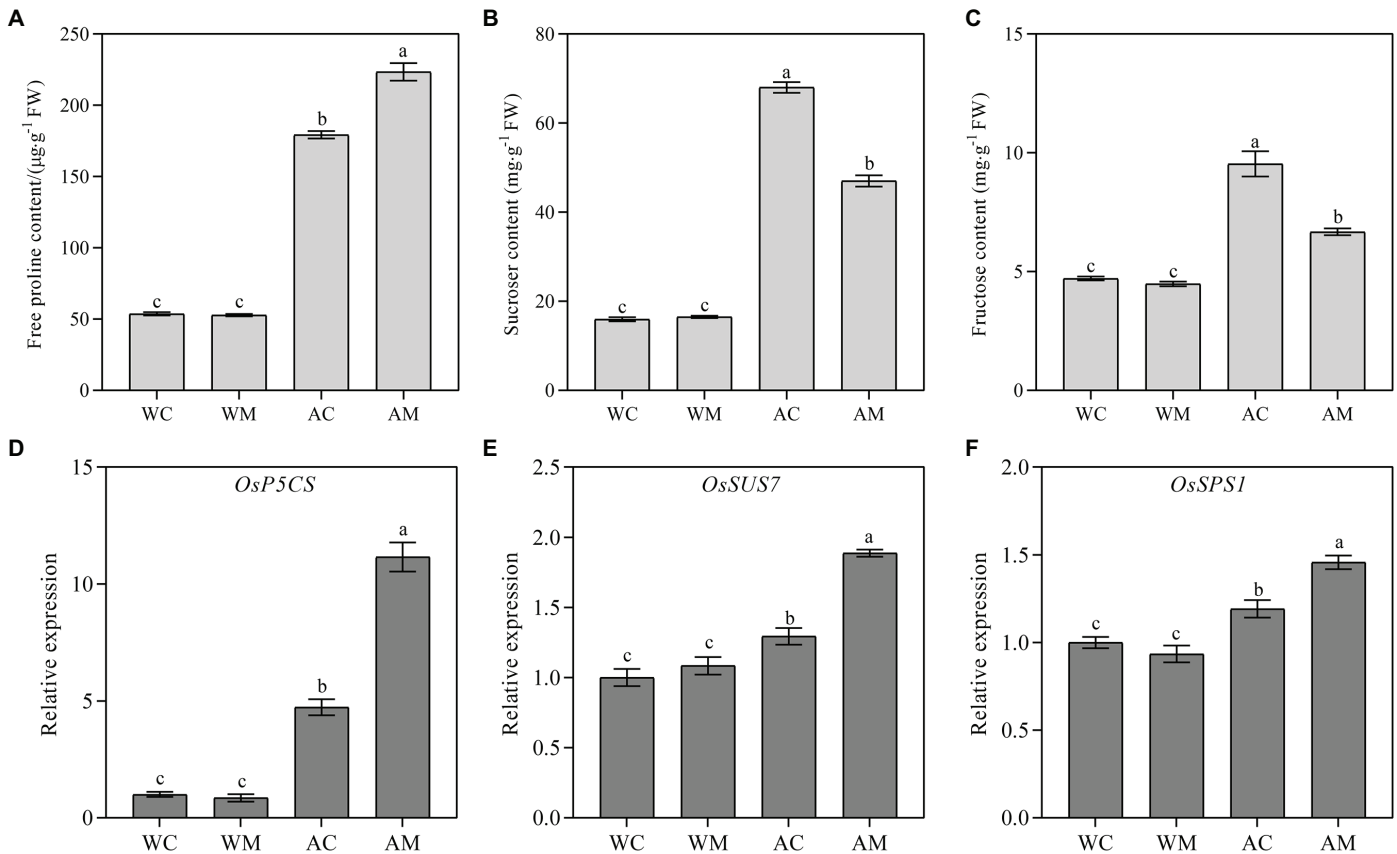
Effects of exogenous MT on proline, sucrose, fructose content, and their related gene expression. **(A)** Proline content; **(B)** Sucrose content; **(C)** Fructose content; and **(D–F)** *OsP5CS*, *OsSUS7*, and *OsSPS1* relative expression level. Data represent means ± SEs of three replicate samples. Different letters denote significant differences (*n* = 3, and *p* < 0.05). WC, control; WM, control pretreated with MT; AC, alkaline stress; and AM, alkaline-stressed plants pretreated with MT.

Free proline, sucrose, and fructose are important osmotic substances that could maintain cell turgor pressure and keep the cell growing continuously. Compared to the control (WC), spraying MT had no remarkable change on proline, sucrose, and fructose contents under normal growth conditions (WM). However, under alkaline stress, MT pretreatment significantly increased proline content and decreased sucrose and fructose contents (Figures 7A–C). Under alkali stress, it was also possible that even when melatonin was sprayed, sucrose and fructose may be further converted into monosaccharides for rice seedling growth.

To reveal the regulatory mechanism of osmotic adjustment substances in rice seedlings, response to alkali stresses with MT pretreatment and the transcript levels of three osmotic adjustment-related genes, *OsP5CS, OsSUS7,* and *OsSPS1,* were checked in rice seedlings under different treatments. The results indicated no difference between WM and WC seedlings in three osmotic adjustment-related gene expression levels. In contrast, the gene expression levels of AM treatment were significantly higher than that of AC treatment in rice seedlings (Figures 7D–F), suggesting exogenous MT mainly increased the expression of *OsP5CS*, which was conducive to the accumulation of more proline, thus alleviating the damages of alkali stress.

### Changes of MT Synthetase Gene Expression Levels

**Figure 8:**
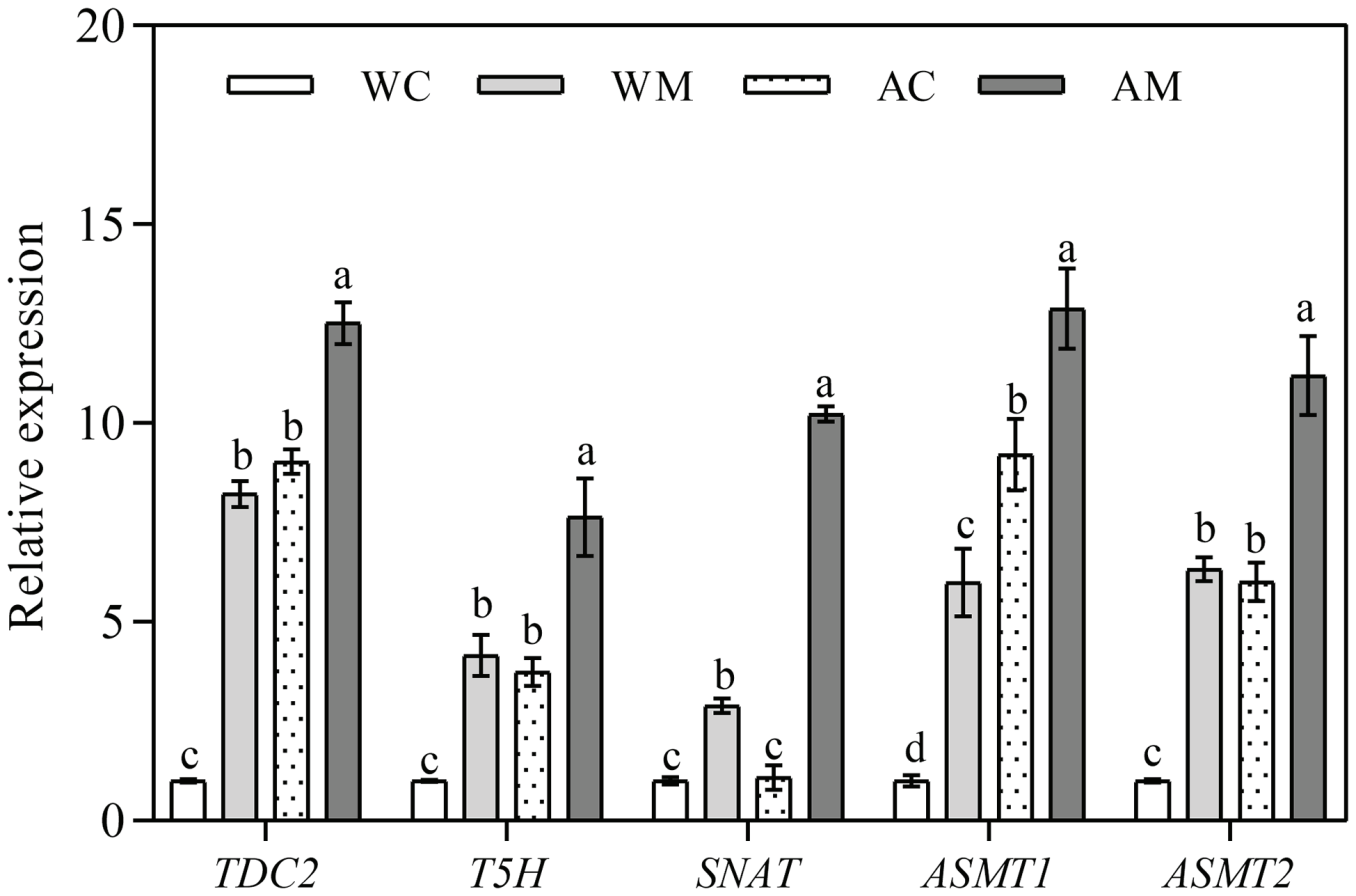
Effect of exogenous MT on expression of melatonin synthesis genes. Data represent means ± SEs of three replicate samples. Different letters denote significant differences (*n* = 3, and *p* < 0.05). WC, control; WM, control pretreated with MT; AC, alkaline stress; and AM, alkaline-stressed plants pretreated with MT.

The results mentioned above showed that exogenous MT could enhance the ability of rice seedlings to resist alkali stress. To further understand whether exogenous MT affected the expression levels of MT synthetase genes in rice seedlings, *TDC2*, *T5H*, *SNAT*, *ASMT1,* and *ASMT2* were selected. Then, their expression levels were determined in different treatments. The results showed that exogenous MT remarkably induced the expression levels of MT synthetase genes in varying degrees under both normal growth conditions and alkaline stress (Figure 8). Among these five genes, the fold change of *SNAT* gene expression levels was the most significant under alkali stress, which adequately verified that *SNAT* acted as one of the important rate-limiting enzyme genes in melatonin synthesis.

### Linear Relationship Analysis of MT Synthetase Genes and Antioxidase Genes

**Figure 9:**
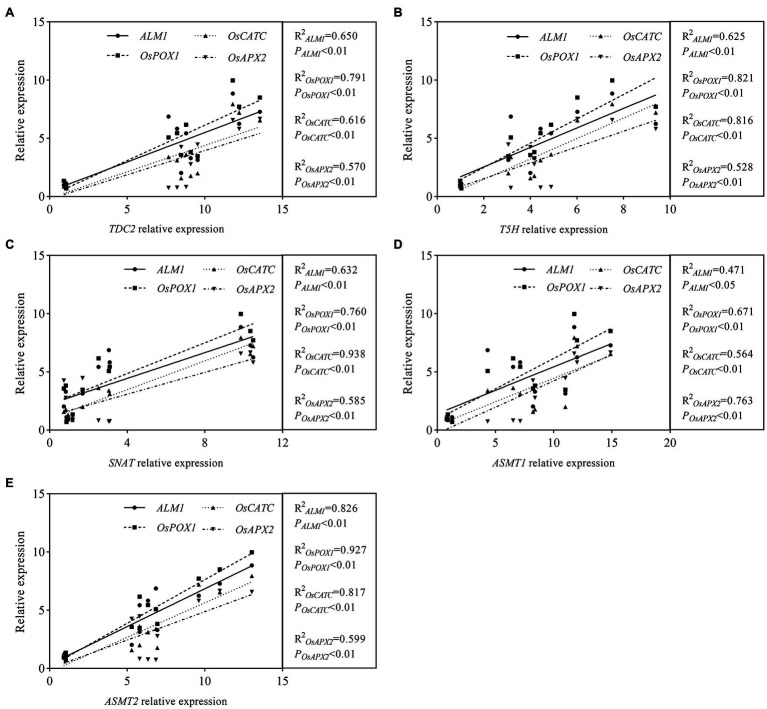
Colinearity analysis of melatonin synthesis genes and antioxidase genes. **(A)** Colinearity analysis of TDC2 and antioxidase genes. **(B)** Colinearity analysis of T5H and antioxidase genes. **(C)** Colinearity analysis of SNAT and antioxidase genes. **(D)** Colinearity analysis of ASMT1 and antioxidase genes. **(E)** Colinearity analysis of ASMT2 and antioxidase genes.

Studies have proven that MT could increase plant tolerance to abiotic stresses by regulating antioxidase gene expression levels and activating antioxidant enzyme activities. To explore whether there is a linear relationship between the expression levels of MT synthetase genes (*TDC2*, *T5H*, *SNAT*, *ASMT1*, and *ASMT2*) and that of antioxidase genes (*ALM1*, *OsPOX1*, *OsCATC*, and *OsAPX2*) in rice seedlings, the linear relationship analysis between five MT synthetase genes and four antioxidase genes was conducted based on gene’ relative expression levels. The results showed that *F* values between *TDC2* and antioxidase genes (*ALM1*, *OsPOX1*, *OsCATC*, and *OsAPX2*) were 18.56, 37.88, 16.04, and 13.26 (Data were not shown in Figure 9A), and *R*^2^ value was displayed as 0.650, 0.791, 0.616, and 0.570, respectively (Figure 9A). A similar result occurred between each of the other four melatonin synthetase genes (*T5H*, *SNAT*, *ASMT1*, and *ASMT2*) and four antioxidase genes (*ALM1*, *OsPOX1*, *OsCATC*, and *OsAPX2*; Figures 9B–E), suggesting that the expression level of MT synthetase genes and antioxidase genes exist in a linear relationship.

### Establishment of Cell Metabolism Model for the Alleviating Effect of Exogenous MT on Rice Seedlings Effected Alkaline Stress

**Figure 10:**
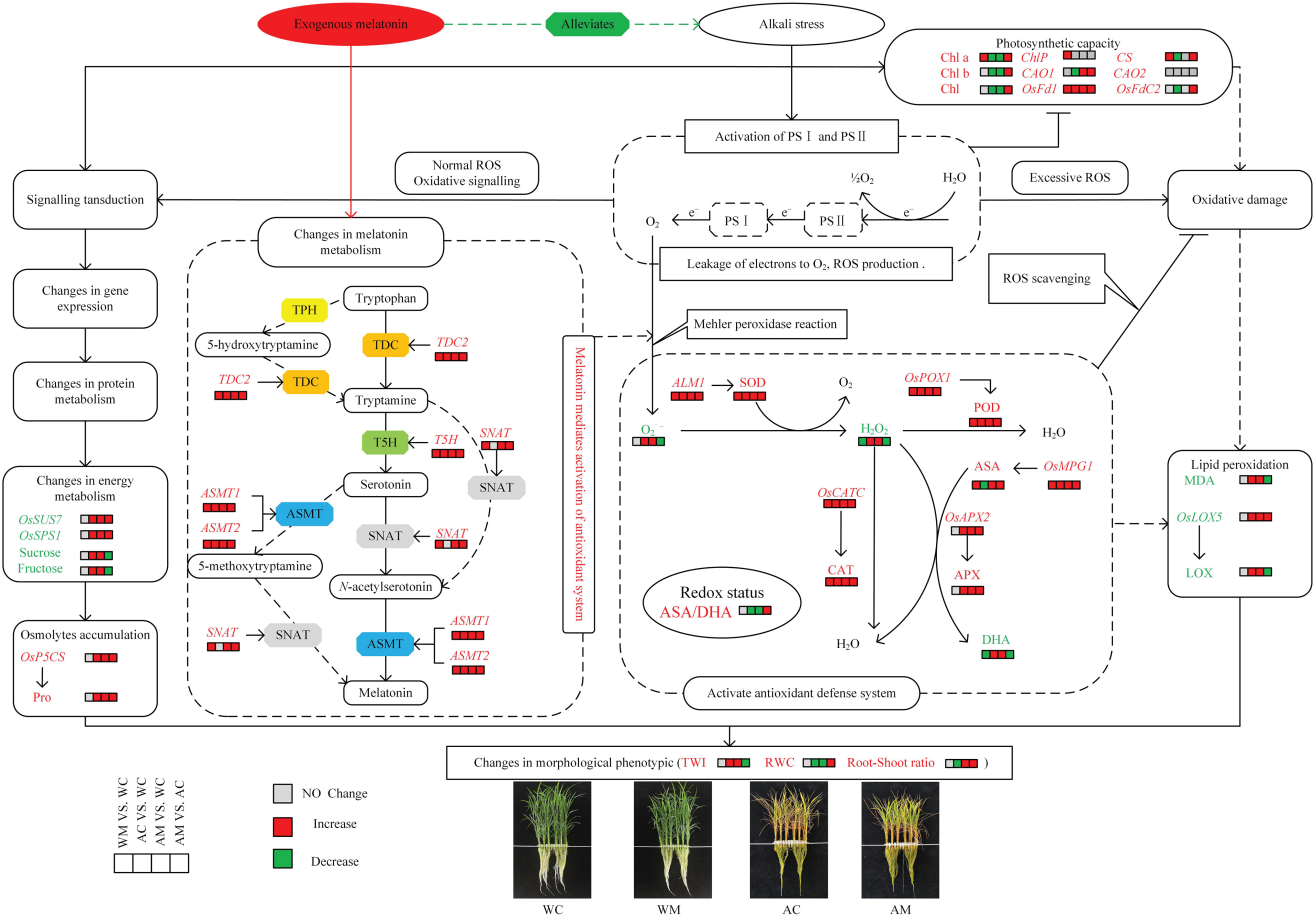
Cell metabolism model of exogenous MT on alleviating effects of rice seedlings under alkaline stress. The solid (dotted) black arrows and the solid (dotted) terminated lines represent direct (indirect) positive influence and direct (indirect) negative influence, respectively. The red and green boxes represent a significant increase and decrease in the measured indicator content in a certain MT treatment, respectively. The gray boxes indicate no significant changes in the measured indicator content between non-stressed and stressed conditions.

A theoretical model was drawn based on the key traits of morphology, physiology, and biochemistry to better understand the alleviating effect of exogenous MT on rice seedlings affected by alkaline stress. The transcription level of some genes related to physiological and biochemical metabolism in rice seedlings under four treatments (WC, WM, AC, and AM; Figure 10) was also studied. Under normal conditions, exogenous MT increased the chlorophyll content and activated the activities of SOD, POD, CAT, and ASA. However, it decreased the contents of H_2_O_2_ and DHA simultaneously. Furthermore, the expression levels of chlorophyll synthesis-related genes (*Chlp*, *CS*, and *OsFd1*), antioxidant synthesis genes (*ALM1*, *OsPOX1*, *OsCATC*, *and OsMPG1*), and MT synthetase genes (*TDC2*, *T5H*, *SNAT*, *ASMT1*, and *ASMT2*) were upregulated in rice seedlings. However, in the case of alkali stress, there is a decrease in chlorophyll content (Chl*a*, Chl*b*, and Chl) and the downregulation of chlorophyll synthesis-related genes (*CS*, *CAO1*, and *OsFdC2*) expression levels, and yet increase of ROS (O_2_^·–^ production rate and H_2_O_2_ content) in rice seedlings. A series of defense signaling cascades must be activated to resist alkaline stress.

Further, some gene expression, protein, and energy metabolism need to be adjusted in rice seedlings. For instance, the expression of *OsP5CS*, *OsSUS7,* and *OsSPS1* was upregulated, which contributed to the accumulation of osmotic adjustment substances such as sucrose, fructose, and free proline. It is well known that ROS production is an early defense signal of rice and participates in the transmission of oxidative signals. However, ROS overproduction triggered oxidative damage and changed LOX activity and increased MDA content. The occurrence of oxidative signal transduction or oxidative damage mainly depends on the balance between ROS generation and scavenging. The expression levels of several antioxidase genes (*ALM1*, *OsPOX1*, *OsCATC*, *OsAPX2, and OsMPG1*) were significantly upregulated, and the activities of SOD, POD, CAT, and APX were significantly increased and inversely decreased ASA/DHA ratio in rice seedlings to balance oxidative damage. In the meantime, alkaline stress induced the expression levels of melatonin synthetase genes, including *TDC2*, *T5H*, *ASMT1*, and *ASMT2*.

Further, exogenous MT pretreatment was beneficial to upregulate the transcriptional levels of all melatonin synthetase genes, which might affect the synthesis of melatonin precursor and melatonin in rice seedlings under alkaline stress. The accumulation of melatonin contributed to the removal of ROS and balancing oxidative damage. In this study, MT effectively alleviated the damage caused by alkaline stress, specifically embodied in increasing the chlorophyll content (Chl*a*, Chl*b*, and Chl) and regulating the expression levels of chlorophyll synthesis-related genes (*CS*, *CAO1*, *OsFd1*, and *OsFdC2*). It also activates and regulates SOD, POD, CAT, and APX activity and increases the ASA/DHA ratio by upregulating the expression levels of antioxidase genes such as *ALM1*, *OsPOX1*, *OsCATC*, *OsAPX2,* and *OsMPG1*. However, it decreases MDA content and LOX activity by changing *OsLOX5* gene expression level and adjusting energy metabolism and Osmolytes content (sucrose, fructose, and free proline) by regulating the expression levels of *OsP5CS*, *OsSUS7*, and *OsSPS1.* Ultimately, it was reflected phenotypically that exogenous MT could effectively alleviate the damage of alkaline stress on rice by decreasing leaf tip wilt index (TWI) and maintaining higher leaf RWC and increasing root-shoot ratio.

## Discussion

When the plants under saline-alkali stress, the root first perceives the stress signaling and gradually transmits it to the aboveground part, which ultimately affects plant growth (An et al., 2021). Salinity and alkaline stress led to the preferential decomposition of chlorophyll and chloroplast, resulting in leaf yellowing, premature senescence, and decreased photosynthesis (Zhu et al., 2019). Exogenous MT could slow down rice senescence under salt stress (Liang et al., 2015). In the results of this study, the application of exogenous MT effectively alleviated the premature senescence of rice leaves under alkaline stress. This alleviation of the premature senescence of rice leaves was manifested as the increase in the number of green leaves in rice seedlings (Figure 1A), the decrease in TWI, the increase in leaf water content, and lastly, the root-shoot ratio (Figure 1). Chlorophyll is necessary for photosynthesis, and high chlorophyll content is conducive to utilizing light energy. However, salt and alkaline stress degrades chlorophyll in plant leaves. Yan et al. (2021b) proved that exogenous MT alleviated salt stress by improving leaf photosynthesis and chlorophyll content. In the results of this study, under normal conditions, exogenous MT upregulated the expression levels of *ChlP*, *CS*, *OsFd1,* and *OsFdC2* and increased the content of chlorophyll a. Under alkaline stress, MT pretreatment increased the contents of chlorophyll a, chlorophyll b, and total chlorophyll in rice leaves. The expression levels of chlorophyll synthesis-related genes (*CS*, *CAO1*, *OsFd1*, and *OsFdC2*) were also upregulated (Figure 2). Wang et al. (2022) also found that MT pretreatment induced upregulation of several chlorophyll synthesis genes to alleviate NO_2_ stress. In addition, melatonin may reduce chlorophyll degradation by inhibiting the expression of chlorophyll degradation key enzyme gene *PAO* (Weeda et al., 2014). Therefore, MT pretreatment may increase the chlorophyll content of rice seedlings under alkaline stress by upregulating chlorophyll synthesis genes and downregulating chlorophyll degradation genes, thereby delaying senescence and increasing photosynthetic rate.

Salinity-alkalinity stress leads to the imbalance between photosynthetic electron transport and the Calvin cycle. Hence, there is a decrease in electron carriers in chloroplasts and mitochondria, leading to the transfer of electrons from cytochrome to oxygen molecules and the production of excessive ROS. The excessive production of ROS leads to oxidative stress, which damages the cytoplasmic membrane and leads to cell damage and death (Mittler et al., 2004). LOX is an oxidation and lipid degradation enzyme that catalyzes lipid peroxidation (Doderer et al., 1992). MDA is a lipid peroxidation product and is associated with ROS accumulation (Puyang et al., 2015). Hence, LOX and MDA reflect the damage of cell membrane. In this study, after alkaline stress, ROS (O_2_^·–^ and H_2_O_2_) content in rice was over-accumulated (Figure 3), LOX activity and MDA content were significantly increased (Figure 4). However, the application of exogenous MT pretreatment reduced LOX activity, ROS, and MDA content under alkaline stress (Figures 3, 4). Similar results were also found in already published studies of cucumber (Wang et al., 2016) tomato (Jahan et al., 2020), and rice (Chen et al., 2021). Exogenous MT pretreatment reduced the contents of H_2_O_2_, O_2_^·–^, and MDA in cucumber, tomato, and rice under salt stress. In addition to regulating plant development, MT is known as an antioxidant. As reported in this study, MT may be directly involved in ROS scavenging and inhibiting lipid peroxidation under alkaline stress (Figures 3, 4), thereby maintaining ROS balance in rice seedlings affected by alkaline stress.

Antioxidant enzymes and non-enzymatic antioxidants are the most direct and effective ways to scavenge ROS in plants (Apel and Hirt, 2004). In this study, antioxidant enzymes (SOD, POD, CAT, and APX) and DHA were accumulated in large quantities under alkaline stress; however, antioxidant (ASA) had no significant change. Similarly, the expression level of antioxidase genes (*ALM1*, *OsPOX1*, *OsCATC*, and *OsAPX2*) and antioxidant-related genes (*OsMPG1*) were induced under alkaline stress (Figures 5, 6). Under abiotic stress, MT reduces ROS accumulation by activating antioxidant enzymes and increasing the expression of related genes (Al-Huqail et al., 2020). Similar to the results found in this study. For example, MT pretreatment significantly enhanced SOD, POD, and CAT activities under normal conditions, increased ASA content, and decreased DHA content. Meanwhile, the expression levels of *ALM1*, *OsPOX1*, *OsCATC,* and *OsMPG1* were significantly increased, indicating that MT can improve its efficiency as an antioxidant by regulating the activity of antioxidant enzymes (Nawaz et al., 2018). Under alkaline stress, MT pretreatment increased the activities of SOD, POD, CAT, and APX. It also increased ASA content and decreased DHA accumulation; upregulated the expression of *ALM1*, *OsPOX1*, *OsCATC*, *OsAPX2,* and *OsMPG1*, thereby enhancing the tolerance of rice to alkaline stress. Similar results were also observed in rice (Yan et al., 2021a) and maize (Chen et al., 2018). Therefore, MT may increase the activity of antioxidant enzymes (SOD, POD, CAT, and APX) and antioxidant (ASA) content, and reduce DHA content by upregulating antioxidase genes and antioxidant-related genes and then reducing excessive ROS accumulation. However, Zhang et al. (2016) found that exogenous MT inhibited dark-induced leaf senescence mainly by activating SOD-CAT antioxidant pathway and slowing down chlorophyll degradation, but did not change the APX activity of perennial ryegrass under dark induction, and reduced the content of non-enzymatic antioxidants such as ASA. This is contrary to the results of this study, indicating that MT has different physiological mechanisms in alleviating alkali stress in rice seedlings.

Proline, sucrose, and fructose play important roles in plant growth, development, and resistance to abiotic stresses (Kishor et al., 2015; Sharma et al., 2020). Jahan et al. (2019) confirmed that MT pretreatment could improve proline content and *P5CS* expression level of tomato under high-temperature stress. Similarly, in this study, proline, sucrose, and fructose content increased significantly under alkaline stress. Exogenous MT could further improve the proline content and the relative expression level of *OsP5CS* under alkaline stress (Figures 7A,D). Yan et al. (2021a) found that exogenous MT treatment increased the sucrose content of rice under salt stress. However, in our study, exogenous MT treatment reduced sucrose and fructose contents in rice seedlings under alkaline stress (Figures 7B,C). Still, it upregulated the expression of sucrose synthesis-related genes *OsSUS7* and *OsSPS1* (Figures 7E,F). It may be because MT pretreatment accelerated the transformation of sucrose and fructose in plants (Zhao et al., 2016), resulting in the decrease of sucrose and fructose content compared with that under alkaline stress. Therefore, exogenous MT may maintain the alkaline tolerance of rice by increasing the Pro content in plants and accelerating the transformation of sucrose and fructose *in vivo*.

It is well known that SNAT and ASMT are recognized as melatonin biosynthesis rate-limiting enzymes in melatonin synthase. Overexpression of SNAT and ASMT synthesis genes can significantly enhance plant tolerance to stress. The overexpression of *SlSNAT* gene in tomatoes significantly increased the endogenous melatonin level, reduces the accumulation of ROS and *Fv*/*Fm* level, and improved tomato’s heat tolerance (Wang et al., 2020). In Arabidopsis, overexpression of *MzASMT* increased endogenous melatonin levels, decreased ROS, and enhanced tolerance to drought stress (Zuo et al., 2014). TDC and T5H have high catalytic efficiency, while SNAT and ASMT are rate-limiting enzymes in melatonin synthesis. Therefore, melatonin synthesis precursors such as serotonin are higher under stress conditions than melatonin (Tan and Reiter, 2020). Because of plant cells may absorb exogenous MT to increase intracellular MT content, and the increase of endogenous MT content can activate the feedback effect of plant cells, thereby weakening the synthesis rate of endogenous MT, further research is needed to distinguish the source of MT content in rice seedlings. In this study, exogenous MT pretreatment significantly increased the expression levels of *TDC2*, *T5H*, *SNAT*, *ASMT1,* and *ASMT2* under normal conditions. Under alkaline stress, *TDC2*, *T5H*, *ASMT1,* and *ASMT2* were upregulated, while *SNAT* expression did not change, which may be due to *SNAT* as a rate-limiting enzyme in melatonin synthesis. Exogenous MT pretreatment further improved the expression levels of *TDC2*, *T5H*, *SNAT*, *ASMT1,* and *ASMT2* under alkaline stress (Figure 8). It is suggested that exogenous MT can induce the upregulation of melatonin synthesis genes, which may enhance the synthesis rate of endogenous MT and improve rice’s alkaline tolerance.

Melatonin enhances plant tolerance to various stresses by indirectly activating antioxidant enzymes (Li et al., 2017; Nawaz et al., 2018; Al-Huqail et al., 2020). Therefore, we conducted a linear relationship analysis of MT synthetase and antioxidase genes. The results showed that MT synthesis genes (*TDC2*, *T5H*, *SNAT*, *ASMT1*, and *ASMT2*) had an aboriginal linear relationship with antioxidase genes (*ALM1*, *OsPOX1*, *OsCATC*, and *OsAPX2*), suggesting that exogenous MT induces the expression of MT synthesis genes, thereby increasing the content of endogenous melatonin. Further, MT was involved in regulating the expression of antioxidase genes, affecting the activity of antioxidant enzymes, and ultimately improving the alkaline tolerance of rice.

Previous studies indicated that there existed crosstalk between MT and NO, as well as between MT and AM fungus in plants (Liu et al., 2015; Yang et al., 2020). This study only proved that exogenous MT alleviated alkaline stress by removing ROS and improving antioxidant capacity in rice seedlings. However, whether other signal molecules such as NO, ABA, and GA has a functional role in regulating rice to alkaline stress response? Whether there are interactive effects of exogenous MT and other signal molecules such as NO, ABA, and GA on alkaline stress tolerance in rice? The underlying mechanism of exogenous MT or combination of MT and ABA (hypothetically) in regulating rice tolerance to alkaline stress requires further research by characterizing MT and/or ABA biosynthesis rate-limiting enzyme genes in transgenic and CRISPR-Cas9-edited rice.

In summary, our results first demonstrated that exogenous MT upregulated the transcriptional level of MT synthesis genes and proved a line relationship between MT synthesis genes and antioxidant enzyme synthesis genes (Figure 9). This study provides a new insight that exogenous MT alleviated rice alkaline stress by mainly regulating endogenous MT metabolism-controlled ROS metabolism.

## Data Availability Statement

The original contributions presented in the study are included in the article/supplementary material, and further inquiries can be directed to the corresponding author.

## Author Contributions

CL supervised and designed the experiments. XL guided the research and wrote the manuscript. XL, WM, YS, LT, PL, TM, and YZ performed the experiments and data analysis. All authors have read and approved the final manuscript.

## Funding

This work was supported by the National Natural Science Foundation of China (NSFC; grant no. 32060425), the Natural Science Foundation of Ningxia Province (grant no. 2020AAC03095), and the Postgraduate Innovation Foundation of Ningxia University (grant no. GIP2021-12).

## Conflict of Interest

The authors declare that the research was conducted in the absence of any commercial or financial relationships that could be construed as a potential conflict of interest.

## Publisher’s Note

All claims expressed in this article are solely those of the authors and do not necessarily represent those of their affiliated organizations, or those of the publisher, the editors and the reviewers. Any product that may be evaluated in this article, or claim that may be made by its manufacturer, is not guaranteed or endorsed by the publisher.
